# The New Boarding-Out Order

**Published:** 1911-11-18

**Authors:** 


### THE NEW BOARDING-OUT ORDER.

Even those who have little sympathy with Mr. Burns' general politics agree that as President of the Local Government Board he does admirable work. He is energetic and practical, he sees things for himself, and is not to be lured into the slough of officialism. All who are interested in the children of the State will welcome the new order regarding boarded-out children, which will do much to facilitate the placing of suitable children in an environment, which, even if not ideal, is far more wholesome than the workhouse or even the Poor Law school. There was for some time a tendency to abandon boarding-out because it gave more trouble to the guardians, and also because some boards grudged the fares of visiting guardians. The "Fresh Air funds" which have sprung up of recent years have indirectly brought boarding-out into fashion again by showing how many cottage homes there were where a child could be placed safely and comfortably, and also by breaking down the prejudice of country folks against town-bred children. The success of the fresh-air schemes depends on the co-operation of friends in the country as well as of sympathisers in the towns, and these country friends—many of them ladies—have so far familiarised themselves with the condition and needs of the town waif and the sort of home needed, that they are admirably fitted to undertake the general supervision of children who are permanently boarded out in a country home. It is good to see that the new order is arranged so as to make use of all who will help in the supervision of these children, and especially to utilise the kind offices of women. Formerly it was fixed that there should be at least one woman on every boarding-out committee, but anyone who knows the details of the work will agree that Mr. Burns has done well in increasing the minimum number of women to a third of the total number of members of committee. In many boards of guardians this extension will make no difference, as they had already gone beyond the single female member enjoined upon them, but it will make a rule of what was hitherto only a fortunate exception.

With regard to the composition of the committees further flexibility is permitted. A committee may consist of guardians only or may include other persons as well, while if they think it wise the guardians may "enter into administrative arrangements with persons acting for the purpose independently of the guardians." If the children are boarded out beyond the Union to which they are chargeable, the guardians may enter into arrangements with an independent committee, or avail themselves of local knowledge by arranging with a committee appointed by the guardians of the Union where the child is sent to live. Thus every available form of help and guidance can be brought to bear on the bringing-up of the children. But one thing is specially enjoined—that every child must be visited at least once in six weeks by a woman member of the boarding-out committee or by an appointed woman visitor, and it is recommended that where a paid appointed visitor is employed her visits should alternate with those of a member of the committee. It is not considered necessary that the visitor should give her whole time to the work, and the services of the district nurse or the visitor under the Infant Life Protection Act may be utilised to see to boarded-out children, while the visitor is further empowered to go to the homes of persons in receipt of out-relief who have children dependent upon them, and ascertain the condition of the children. It is pitiful but true that there are children living with a parent who are worse off than orphans. Relief is given for them, and is not properly spent upon them. Hitherto it has been difficult to deal with these cases except when the mother or other guardian was absolutely bad, dirty, or drunken, or immoral, but now the same standard of wholesomeness and decency which the orphaned or deserted children enjoy can be enjoined for the benefit of children living with relations. It is to be hoped that the woman visitors will take full advantage of the powers entrusted to them. There are many people who can put in a decent appearance before the guardians whose homes and habits will not satisfy a woman visitor.

It is a question whether, in face of the rise in the prices of necessaries, it might not be advisable to increase the allowance paid for boarded-out children. The maximum is five shillings per week. This is more than enough for a young child, especially in a country place where milk and vegetables are cheap, but it is doubtful if it is sufficient for a healthy growing boy or girl of ten or twelve. It will cover the cost of food undeniably, but the foster-parents are not to be blamed if they expect to make a small profit out of the children who share their home. No one takes "paying guests" for the mere love of it—though perhaps boarded-out children may get more done "for love" than children of a higher grade. But as the standard of accommodation demanded for them, and rightly demanded, is perhaps above the normal standard of cottages, in respect of privacy as well as comfort, some remuneration must be given for it. And a maximum of five shillings a week leaves little margin for extra rent. And it should be remembered to what an extent the cost of all necessaries varies in different places. What is an ample payment in one place may be so unremunerative in another that the children's food has to suffer to leave any margin for the foster-parents. To decide what is a sufficient payment without being an excessive one is a matter for the local committee to say, while the guardians of the Union to which a child is chargeable will assuredly inform themselves as to the average wage in any locality to which they may send any of their wards, and be guided to some extent by that in the sum they are prepared to give. And with the more frequent inspection by women visitors which we are promised, practical information on a subject which is so peculiarly within woman's province should be available, and should help to reduce anomalies and increase efficiency in the management and bringing-up of boarded-out children.

